# The Lord Is Risen

**Published:** 1890-04-05

**Authors:** 


### Words of Consolation.

### THE LORD IS RISEN.

The dreadful sacrifice has been accomplished. The dead Christ has been deposited in the Tomb, and the disciples have gone to their homes, sick, weary, and desponding, at the disappointment of all their hopes. How sad and dreary must life have seemed to them! He who for three years had been their Teacher, Guide, and constant companion, He to whom they had looked for deliverance from their Roman masters, had suffered an ignominious death, and disappeared for ever (as they supposed) from their sight. The women who had ministered to His necessities were equally ignorant. Like the disciples they could not grasp that Christ's death was the only road to glory. So they rested on the Sabbath according to the commandment, and came early in the morning of the first day of the week, bringing with them sweet spices to embalm His body. But they find the tomb is empty! In their weak faith and slowness of heart they would rather have found it full. They knew not that the grave could not contain Him, death could have no dominion over Him. Christ had risen, leaving the grave clothes and the napkin, as the only signs of what the tomb had once contained. The sorrowing women, full of doubt and anxiety, wondered how they should roll away the great stone from the cave's mouth. To their amazement it was already gone. An Angel had come down from Heaven and, with an earthquake, made their way clear before them, and their suffering hearts were cheered by the message, "He is not dead, He has risen; go tell His disciples."

Although our Lord had been withdrawn from His disciples for a season, yet He soon showed Himself to them all, separately or in twos and threes, according to their several necessities. To Mary Magdalen, whose love, strong as death, had brought her first to the tomb, He first vouchsafed to be known. Her sorrowing heart went out in the bitter cry, "They have taken away my Lord." With compassionate love He calls her by name, and, immediately recognizing Him, she falls at His feet in reverent thankfulness.

The two disciples on their road to Emmaus were reasoning with themselves whether Jesus was the Christ or not. Their bitter disappointment at His death burst forth, when our Saviour joined them in their walk. "We trusted that it had been He which should have redeemed Israel." Then our Lord showed them from the Scriptures what Christ should suffer, and as they pressed Him to remain with them, the day being far spent, He made Himself known unto them in the breaking of bread.

